# Commentary: Why sprint interval training is inappropriate for a largely sedentary population

**DOI:** 10.3389/fpsyg.2015.01359

**Published:** 2015-09-07

**Authors:** Fabrício B. Del Vecchio, Paulo Gentil, Victor S. Coswig, David H. Fukuda

**Affiliations:** ^1^Department of Performance and Human Metabolism, Superior School of Physical Education, Federal University of PelotasPelotas, Brazil; ^2^Department of Physical Education, University of BrasiliaBrasilia, Brazil; ^3^Department of Exercise Physiology, Institute of Exercise Physiology and Wellness, University of Central FloridaOrlando, FL, USA

**Keywords:** exercise psychology, sprint interval training, exercise intensity, behavior change, feeling states, exercise adherence

In this commentary, we explain why the recent manuscript by Hardcastle et al. (2014), which conveyed the opinion that sprint interval training (SIT) is inappropriate for sedentary individuals, may be misguided and propose an alternate view on this issue. Specifically, the main disagreements to consider involve reduced pleasure, self-esteem, adherence, and motivation with SIT, as well as the potential complexities involved with controlling exercise intensities.

Minimal doses of exercise for health are somewhat limited within exercise recommendations (Garber et al., 2011) which tend to gravitate toward moderate intensity continuous exercise (MICE). However, it appears that 150 min/week of MICE is insufficient for weight loss/regain and to influence obesity-related risk factors (Church et al., 2009). Thus, interval-based training at varying intensities is advocated to address health and disease (Gibala et al., 2012). With ~31% of the world's population being sedentary (Hallal et al., 2012), those who aim to improve previously mentioned health goals, SIT, which is a specific type of high-intensity intermittent training (HIIT; Buchheit and Laursen, 2013), may be an advantageous exercise strategy (Del Vecchio et al., 2013).

To advocate for decreased pleasure with increasing intensity, Hardcastle et al. (2014) employ a review article that at no point mentions HIIT or SIT but primarily focused on continuous exercise at ~85% of VO_2_ reserve (Ekkekakis et al., 2011). In a direct comparison of single MICE or HIIT sessions, Oliveira et al. (2013) observed greater ratings of perceived exertion during HIIT, but no difference in physical activity enjoyment between the two types of exercise. In addition to the lack of reference to SIT or HIIT in the currently discussed Opinion Article (Hardcastle et al., 2014), the authors employ an “invited paper” (Parfitt and Hughes, 2009) which focuses on self-selected exercise intensity and self-regulation to support the notion that “enjoyment is also a predictor of exercise adherence and most people do not enjoy high intensity exercise.” Contrary to the suggestions, in a controlled trial, HIIT was shown to be more enjoyable than MICE (Bartlett et al., 2011). Similar results were found in varying populations (Crisp et al., 2012; Jung et al., 2015; Martinez et al., 2015), however, long-term evaluation is still needed.

It is our opinion that the motivation provided by positive health improvements and the time-efficiency of SIT/HIIT exceeds their potential aversive effects. Moreover, the assumption that these protocols have low adherence is not confirmed, with studies in elderly individuals showing a preference for interval protocols (Guiraud et al., 2011) and lengthy training studies (up to 9 months) reporting adherence greater than 90% with HIIT in obese participants and people with joint disorders (Gremeaux et al., 2012; Bressel et al., 2014). The only study cited by Hardcastle et al. (2014) to question adherence to intense protocols is by Perri et al. (2002) which involved the comparison of two continuous exercise sessions carried out between 40–55% and 65–75% of HRreserve. In fact, the results of this study highlight potential issues with current MICE recommendations, including decreased adherence and limitations with regard to training volume using selected intensities for steady-state exercise. Interestingly, results from a systematic review showed that 12mos of MICE resulted in less than 2 kg of weight loss (Avenell et al., 2004), while others have advocated the use of HIIT for improvements in body composition (Boutcher, 2011).

Further, SIT has shown to improve motivation, particularly with regard to appearance and maintenance of body mass, as well as quality of life scores in elderly sedentary people (Knowles et al., 2015). Contradicting the assumptions made by Hardcastle et al. (2014), results from a randomized controlled trial showed that 6 weeks of SIT lead to improvement in the perception of health and mood of sedentary women (30–65 years) at risk for metabolic syndrome (Freese et al., 2014).

The studies used to convince the reader that SIT is strenuous and can increase feelings of low self-esteem, potential failure, and incompetence tended to address generic issues and did not specifically involve SIT or HIIT (Hein and Hagger, 2007; Lindwall et al., 2011). Furthermore, self-discipline and self-regulation, presented as necessary factors to achieve success with SIT, are essential for any behavior change. Thus, engagement in exercise for health is a behavioral decision.

Additionally, the sense of self-esteem, motivation, and competence is relative and can be enhanced by health professionals, as we believe that few people should perform exercise without supervision or guidance with regard to medical clearance, gradual progression, and appropriate monitoring. In this context, the exercise intensity is relative to the individual's current health and emotional status. Often sedentary or obese people and individual's with medical restrictions, have such low physical fitness that it would be impossible to conduct MICE. For example, a person with COPD, if a 30 min exercise is recommended, should exercise at 2.4–3.5 km/h (Rugbjerg et al., 2015), which may results in complications, including, but not limited to, joint pain, diaper rash, and general discomfort as reported by obese individuals during this type of training.

Hardcastle et al. (2014) argue that intensity control during SIT is complex. However, proper control of MICE requires expensive and complex equipment to quantify intensities, such as heart rate monitors, global positioning systems, and/or devices to report external loads (speed, load, inclination, etc.). In contrast, the interval-training model proposed by Tabata et al. (1996) can be conducted using minimal equipment, with physiological adaptions equivalent to those obtained with MICE (McRae et al., 2012). Regarding the use of extended rest/recovery periods between exercise intervals, SIT models are flexible and Matsuo et al. (2014) reported superior results using a protocol lasting only 7 min compared to a 45 min MICE session, further highlighting the time efficient nature of this approach.

Hardcastle et al. (2014) should be commended for potentially furthering the research agenda surrounding the beneficial effects of SIT. In closing, it is should be recognized that the development of training programs should not be limited to a single exercise methodology (Del Vecchio et al., 2013), and that, in addition to SIT and MICE, other modes, including progressive strength training and leisure/recreational activities, should also be utilized in the sedentary population.

## Conflict of interest statement

The authors declare that the research was conducted in the absence of any commercial or financial relationships that could be construed as a potential conflict of interest.
